# Neuregulin Promotes Incomplete Autophagy of Prostate Cancer Cells That Is Independent of mTOR Pathway Inhibition

**DOI:** 10.1371/journal.pone.0036828

**Published:** 2012-05-14

**Authors:** Eran Schmukler, Ben Shai, Marcelo Ehrlich, Ronit Pinkas-Kramarski

**Affiliations:** 1 Department of Neurobiology, Tel-Aviv University, Ramat-Aviv, Israel; 2 Department of Cell Research and Immunology, Tel-Aviv University, Ramat-Aviv, Israel; II Università di Napoli, Italy

## Abstract

**Background:**

Growth factors activating the ErbB receptors have been described in prostate tumors. The androgen dependent prostate cancer cell line, LNCaP, expresses the ErbB-1, ErbB-2 and ErbB-3 receptor tyrosine kinases. Previously, it was demonstrated that NRG activates ErbB-2/ErbB-3 heterodimers to induce LNCaP cell death, whereas, EGF activates ErbB-1/ErbB-1 or ErbB-1/ErbB-2 dimers to induce cell growth and survival. It was also demonstrated that PI3K inhibitors repressed this cell death suggesting that in androgen deprived LNCaP cells, NRG activates a PI3K-dependent pathway associated with cell death.

**Methodology/Principal Findings:**

In the present study we demonstrate that NRG induces autophagy in LNCaP cells, using LC3 as a marker. However, the autophagy induced by NRG may be incomplete since p62 levels elevate. We also demonstrated that NRG- induced autophagy is independent of mammalian target of rapamycin (mTOR) inhibition since NRG induces Akt and S6K activation. Interestingly, inhibition of reactive oxygen species (ROS) by *N*-acetylcysteine (NAC), inhibited NRG-induced autophagy and cell death. Our study also identified JNK and Beclin 1 as important components in NRG-induced autophagy and cell death. NRG induced elevation in JNK phosphorylation that was inhibited by NAC. Moreover, inhibitor of JNK inhibited NRG-induced autophagy and cell death. Also, in cells overexpressing Bcl-2 or cells expressing sh-RNA against Beclin 1, the effects of NRG, namely induction of autophagy and cell death, were inhibited.

**Conclusions/Significance:**

Thus, in LNCaP cells, NRG-induces incomplete autophagy and cell death that depend on ROS levels. These effects of NRG are mediated by signaling pathway that activates JNK and Beclin 1, but is independent of mTOR inhibition.

## Introduction

Prostatic carcinoma is one of the most common male cancers. Prostate cells growth is regulated by hormones, growth factors and their respective receptors. Among the most frequent group of receptors implicated in human cancers is the ErbB subfamily of receptor tyrosine kinases [1], [2], [3]. This family includes four receptors ErbB-1-ErbB-4. Whereas ErbB-1 receptor (known as epidermal growth factor receptor, EGFR), is activated by EGF and EGF-like ligands, ErbB-3 and ErbB-4 receptors are activated by NRG/neuregulin isoforms and ErbB-2 receptor has no known ligand [4]. These receptors are expressed in the prostate epithelium, whereas, ErbB-1 ligands are expressed in the stroma and NRGs are expressed in the stroma and in the basal and secretory epithelium [5].

Activation of ErbB-1 signaling by EGF and EGF-like growth factors plays an important role in prostate cancer cell proliferation and addition of EGF to cultures of prostate cancer cells stimulates their growth [6]. Moreover, ErbB-2 overexpression is a common event that appears to confer a selective advantage to several types of carcinomas including prostate cancer [3], [7]. Normally, ErbB-2 is expressed in prostate epithelial cells [7], [8]. Higher levels of ErbB-2 as compared to normal tissues were observed in prostatic tumors [9], [10]. In addition, overexpression of ErbB-2 and ErbB-3 has been implicated in the neoplastic transformation of prostate cancer [11]. Although the exact role of these oncogenes and growth factors in prostate carcinoma is still unclear, overexpression of ErbB-1 and ErbB-2 has been related to poor prognosis and distant metastasis [12].

Autophagy, a process of regulated turnover of cellular constituents, is important for normal growth control but may be defective in diseases [13], [14]. Under limited nutrients or growth factors conditions, this process is essential to maintain energy production for cell survival [15]. Autophagy can also serve as a mechanism by which cells rid themselves from defective organelles and recycle proteins [16]. On the other hand, autophagy can lead to non-apoptotic type of cell death (type II cell death) playing a role in developmental cell death and death from toxic stimuli [17].

The formation of autophagosomes is controlled by several atg proteins. Atg8 protein (the human homolog is MAP-LC3) is associated with the autophagosomal membrane and serves as a marker for autophagosome formation [18]. Formation of the autophagosome also requires class III phosphatidyl inositol 3-kinase (PI3K) [19]. Autophagy mediated by PI3K depends on interaction of the latter with atg6 protein, of which Beclin 1 is the human homolog [20]. Beclin 1 was shown to act as a tumor suppressor gene by controlling the process of autophagy [21]. Its interaction with the anti-apoptotic protein Bcl-2 [22] inhibits autophagy [23]. Down-regulation of Bcl-2 can apparently promote autophagy [24], suggesting that Beclin 1-mediated autophagy might be inhibited by its interaction with Bcl-2. More recently, several studies identified the Bcl-2 interacting domain in Beclin 1 (a BH3 domain) [25], [26], [27].

Previous studies demonstrated that NRG (ErbB3 and ErbB4 ligand) inhibits growth of the androgen dependent LNCaP prostate cancer cells when cultured in complete medium [28] while in the absence of androgen, NRG induced death of LNCaP cells [29]. Interestingly, PI3K inhibitor (3-methyladenine) that also inhibits autophagy, inhibited NRG-induced cell death, suggesting that NRG may induce autophagic cell death in these cells [29]. In the present study, we addressed the hypothesis that NRG mediates autophagy in LNCaP cells and studied the signaling pathways that mediate NRG induced autophagy and cell death. By using LC3 as a marker, we demonstrate that NRG increases LC3-II levels. However, the levels of p62/SQSTM1 protein, that bind LC3 and is degraded by autophagy [30], were not reduced by NRG treatment, indicating that autophagy induced by NRG is incomplete.

We also demonstrate that inhibition of reactive oxygen species (ROS) by *N*-acetylcysteine (NAC) inhibits NRG-mediated autophagy and cell death. Our results indicate that NRG activates class I PI3K, Akt, mTOR and pS6K pathway, a known autophagy inhibitory pathway, which is not inhibited by NAC. In addition, we demonstrate that NRG induces JNK activation, which is inhibited by NAC. Moreover, JNK inhibitor, Beclin 1 silencing and Bcl-2 overexpression, inhibited NRG-induced autophagy and cell death. Thus, we propose a model by which NRG-induced autophagy and cell death of LNCaP cells involve Beclin 1 and JNK signaling pathways and is independent of PI3K/Akt/mTOR signaling pathway inhibition.

## Results

### NRG Induces Autophagy, which is Inhibited by 3-methyladenine (3-MA) in LNCaP Cells

**Figure 1:**
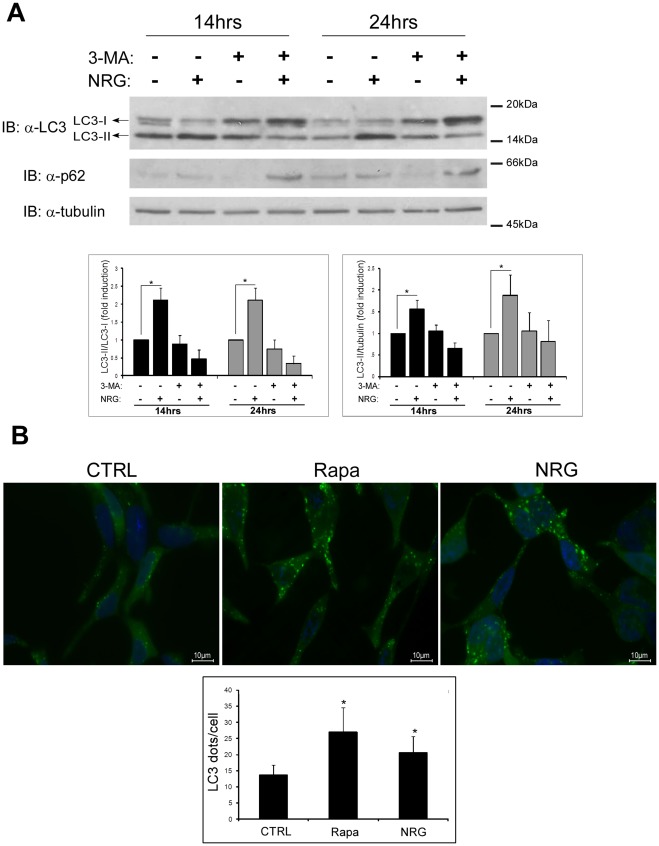
Neuregulin induces autophagy in LNCaP cells. (A) LNCaP cells were treated with 100 ng/ml neuregulin (NRG) in the presence or in the absence of 10 mM 3-methyladenine (3-MA) for the indicated time period. Whole cell lysates were prepared and subjected to an immunoblot analysis with anti-LC3 and anti-p62 antibodies. *Upper panel,* representative results. *Lower panel,* densitometric analysis is presented as fold induction over the control untreated cells (n = 3; means ± S.D; *p<0.05). (B) LNCaP cells stably expressing LC3-GFP were treated with 50 nM rapamycin for overnight or with 100 ng/ml NRG for 5 h. The cells were fixed with 4% paraformaldehyde and nuclei were stained with bisdenzimide (Hoecsht 33258). Following fixation and staining, the cells were photographed using Nikon optical fluorescence microscope Model TE-2000S (60×magnification). *Upper panel*, representative images. *Lower panel*, autophagy was quantified by counting the number LC3 dots per cell using the ImageJ software. The result shown is representative of two independent experiments. 70–80 cells were analyzed per treatment; data presented as mean ± S.D (*p<0.05).

LNCaP is an androgen-responsive prostate carcinoma cell line that expresses the ErbB receptors [29]. Previously, we demonstrated that NRG induces cell death that was inhibited by 3-MA, a PI3K inhibitor [29], and is caspase independent (Not shown and [29]). It was also demonstrated that NRG induces morphological changes in LNCaP cells that were inhibited by 3-MA (Video S1 and [29]). In the present study, we analyzed the effect of NRG on autophagy and cell death of LNCaP cells grown without androgen mimetic. In order to determine autophagy induction, we used LC3 protein as a marker. As shown in Figure 1A, LNCaP cells treated with NRG for 14 h and 24 h, exhibited enhanced conversion of LC3-I to LC3-II, which was inhibited by 3-MA, indicating that indeed NRG induces autophagy in LNCaP cells. To further demonstrate autophagy induction, we used LNCaP cells stably expressing GFP-LC3 expression vector. As shown in Figure 1B, NRG induced enhanced autophagosome formation as reflected by enhanced punctuated staining of GFP-LC3. As a positive control, cells were treated with rapamycin, which also induced autophagy in LNCaP cells (Figure 1B). Thus, LNCaP cells respond to NRG by increased autophagy induction. To further study autophagy induced by NRG, we examined the expression level of p62/SQSTM1. The p62/SQSTM1 protein binds LC3-II and is degraded by autophagy [30]. Surprisingly, NRG treatment did not enhance p62 degradation indicating that autophagy induced by NRG may be incomplete. As a control, cells were treated with Earle's balanced salt solution (EBSS) and the levels of LC3-II and p62 were determined by Immunoblot (Figure S1). As shown, EBSS induced LC3-II elevation and p62 degradation as expected. Of note, 3-MA treatment reduced LC3-II levels in NRG treated cells as well as p62 levels in the absence of NRG. The explanation for these results are yet unknown.

### NRG-induced Autophagy is Incomplete

**Figure 2:**
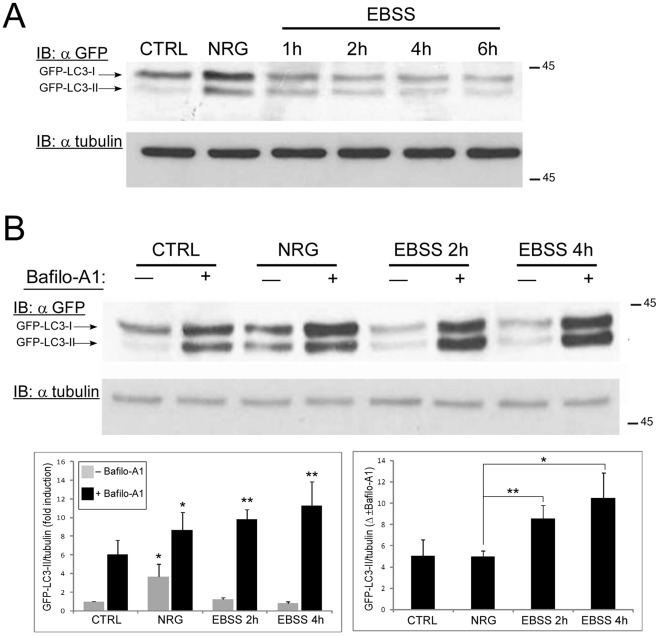
NRG-induced autophagy is incomplete compared to starvation-induced autophagy. (A) LNCaP cells stably expressing LC3-GFP were treated with 100 ng/ml NRG for 24 h or incubated with EBSS medium for the indicated time periods. Whole cell lysates were prepared and subjected to an immunoblot analysis with anti-GFP antibody. (B) LNCaP cells stably expressing LC3-GFP were treated with 100 ng/ml NRG for 24 h or incubated with EBSS medium for 2 and 4 h. Treatments were performed in the presence or absence of 10 nM bafilomycin-A1 (Bafilo-A1). Whole cell lysates were prepared and subjected to an immunoblot analysis with anti-GFP antibody. *Upper panel*, representative blot. *Lower panel*, densitometric analysis is presented as fold induction over the control untreated cells (left graph; *, p<0.05 and **, p<0.02 compared with the control) or as difference between the measured values with or without 10 nM Bafilo-A1 in each group (right graph; *, p<0.05 and **, p<0.02 compared with NRG tretment) (n = 3; means ± S.D).

NRG treatment induces autophagy but also leads to an increase in p62 levels, indicating that the autophagy induced by NRG is incomplete (Figure 1). To further confirm these results, LNCaP cells stably expressing LC3-GFP fusion protein were either treated with NRG for 24 h or incubated with EBSS medium for several hours. Cell lysates were immunoblotted with anti-GFP antibody to detect the levels of GFP-tagged LC3-I and LC3-II. As shown in figure 2A, both EBSS and NRG induce autophagy, as judged by the increase of LC3-II-GFP levels compared to the untreated cells. However, in cells incubated with EBSS, LC3-II-GFP levels decreases over time, while in cells treated with NRG the levels of LC3-II-GFP are relatively high even after 24 h incubation. Furthermore, in the presence of bafilomycin-A_1_ (inhibitor of autophagosome-lysosome fusion) the accumulation of LC3-II-GFP is significantly higher in the EBSS treated cells compared to NRG treated cells (Figure 2B). Taken together, these finding indicate that NRG-induced autophagy is incomplete.

### NRG-induced Autophagy and Cell Death of LNCaP Cells is Inhibited by Reduction of Reactive Oxygen Species (ROS) Levels

**Figure 3:**
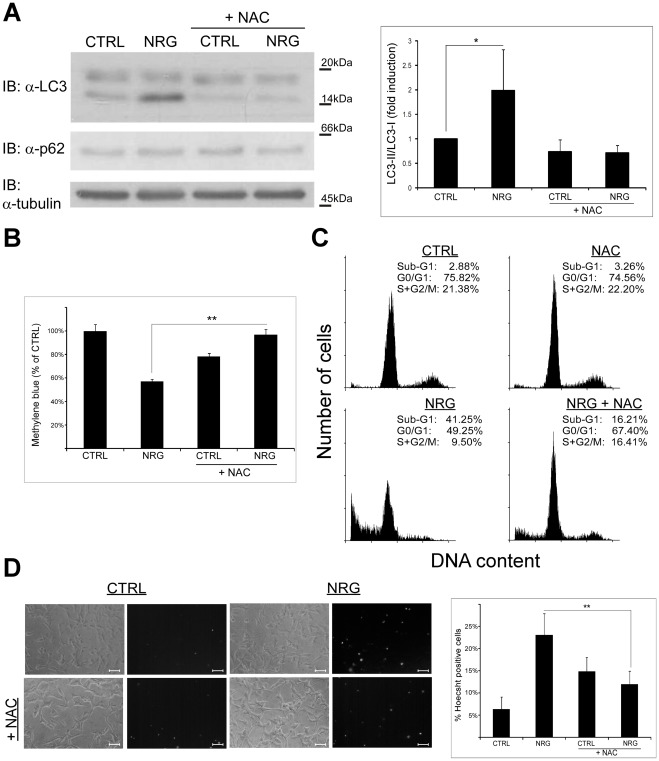
*N*-acetylecysteine inhibits NRG-induced autophagy and cell death. (A) LNCaP cells were treated with 100 ng/ml NRG with or without 10 mM *N*-acetylcysteine (NAC) for 24 h. Whole cell lysates were prepared and subjected to an immunoblot analysis with anti-LC3 and anti-p62 antibodies. *Left panel,* representative results. *Right panel,* densitometric analysis is presented as fold induction over the control untreated cells (n = 6; means ± S.D; *p<0.05). (B) LNCaP cells were tested for cell viability using the methylene blue staining assay. Cells were treated with 100 ng/ml NRG in the presence or in the absence of 10 mM NAC. Methylene blue assay was performed 60 h later. Results are presented as % of control, and are the mean ± S.D of 4–6 determinations (**p<0.0001). This experiment was repeated three times with similar results. (C) LNCaP cells were treated with 100 ng/ml NRG with or without 10 mM NAC. Cells were harvested 60 h later and analyzed for their DNA content by flow cytometry. The percentage of cells at various cell cycle stages is indicated. (D) LNCaP cells were treated with 100 ng/ml NRG in the presence or in the absence of 10 mM NAC for 60 h. The cells were stained with the fluorescent DNA dye bisbenzimide (Hoecsht 33258, 1 µg/ml) to assess the number of dying cells. Following staining, the cells were photographed using Olympus optical inverted phase-contrast microscope Model IX70 (20×magnification; scale bars, 50 micrometer). *Left panel*, representative images. *Right panel*, percentage of dying cells was estimated by counting the number of Hoecsht-positive cells compared to the number total cells in each field (10–15 fields for each treatment, 100–200 cells per field). Results are presented as mean ± S.D (**p<0.0001).

It was previously demonstrated that autophagy induction depends on the formation and accumulation of ROS [23], [31], [32], [33]. Therefore, to characterize the effect of ROS on NRG-mediated autophagy and cell death, LNCaP cells were stimulated with NRG in the presence or in the absence of the general anti-oxidant *N*-acetylcysteine (NAC) [34], and LC3 and p62 levels were determined by immunoblot (Fig. 3A). Pre-incubation with NAC completely inhibited NRG-induced LC3-II elevation, indicating that NRG-induced autophagy is ROS-dependent. Next, we examined whether NAC can protect from NRG-induced cell death. LNCaP cells were pre-treated with NAC with and without NRG treatment, and cell viability was determined using the methylene blue staining assay. As demonstrated in Figure 3B, the presence of NAC prevented the decrease in cell viability induced by NRG. Two additional methods for detection of cell death (Hoecsht dye exclusion assay and flow cytometry) further supported these results. NRG induced enhanced cell death, as evident by the increase in sub-G1 population (Figure 3C) or by the high percentage of Hoecsht-positive cells (Figure 3D). This cell death was markedly inhibited by NAC. Furthermore, NAC treatment inhibited NRG-induced morphological change (S5). Hence, our findings clearly demonstrate that NAC inhibits the LC3-II accumulation, cell death and morphological changes induced by NRG in LNCaP cells.

### NRG-induced LC3-II Elevation and Cell Death in LNCaP Cells is Independent of Akt/mTOR Signaling Pathway

**Figure 4:**
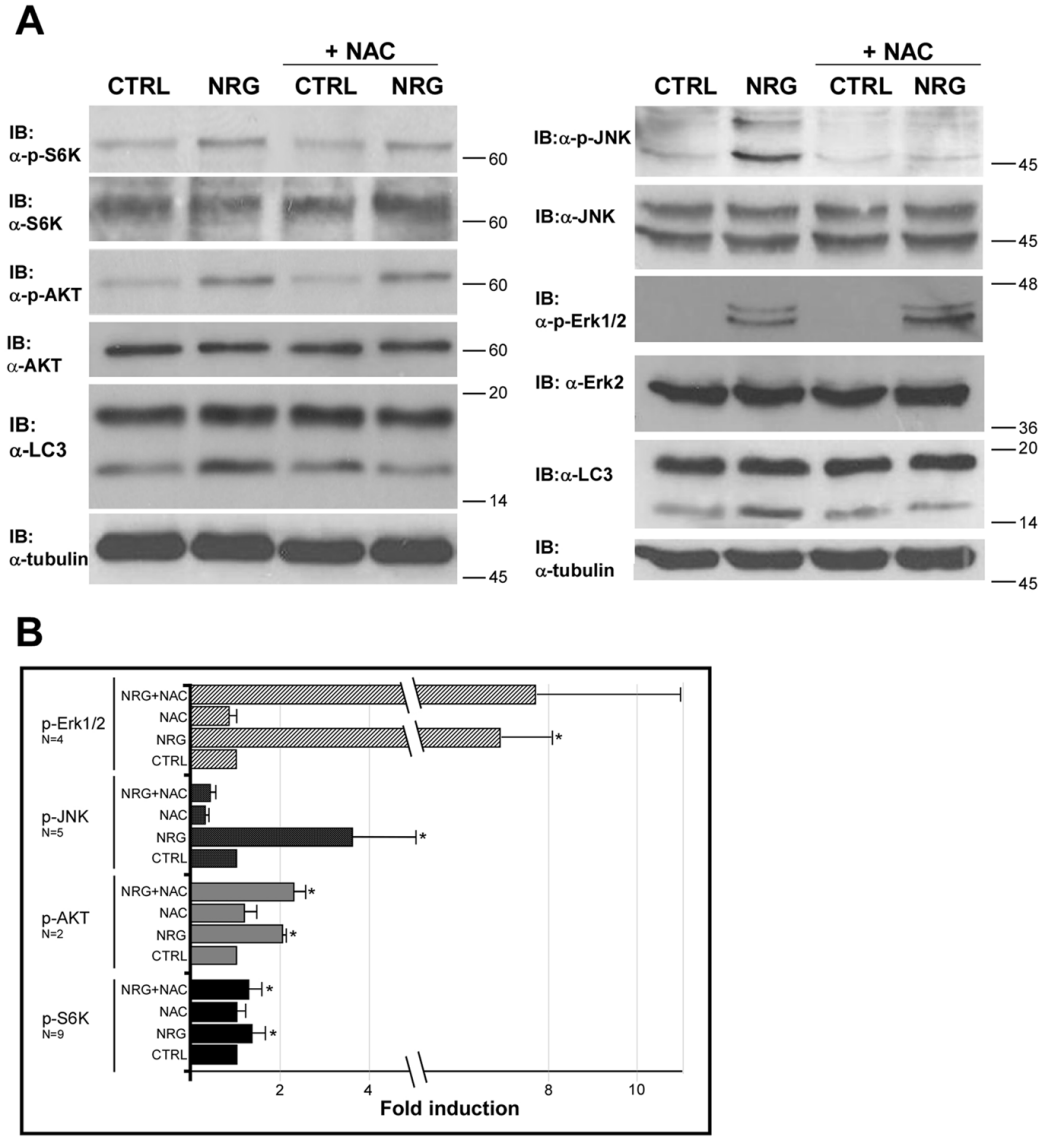
NRG-mediated signaling in LNCaP cells. (A) LNCaP cells were treated with 100 ng/ml NRG with or without 10 mM NAC for 24 h. Whole cell lysates were prepared and subjected to an immunoblot analysis with the indicated antibodies. (B) Densitometric analysis of several repeats is presented as fold induction of the control untreated cells. The means of bands intensity were standardized compared to the total unphosphorylated protein signals (Data are the mean fold induction ± S.D; *p<0.05).

In order to determine the signaling pathway leading to NRG-induced autophagy and cell death, we first examined the Akt/mTOR signaling pathway. LNCaP cells express PTEN mutation that leads to Akt activation [35], [36]. Akt activates mTOR, which is a known negative regulator of autophagy [37], [38]. Thus, it was reasonable to examine the phosphorylation and activation of these proteins. LNCaP cells were treated with NRG for 24 h and the activation of Akt and mTOR were examined using anti-phospho Akt and anti-phospho S6K antibodies. As shown in Figure 4A, basal level of phosphorylated Akt and S6K was observed in the control untreated cells, however, NRG treatment increased the level of phosphorylated Akt and S6K. NAC, which inhibits NRG-induced autophagy, had no effect on the phosphorylation levels of neither Akt nor S6K. These results may suggest that NRG-induced autophagy is independent of mTOR inhibition. To further explore the signaling pathway involved in NRG-induced autophagy in LNCaP cells, we next examined the activation of Erk and JNK, two known mitogen activated protein kinases (MAPKs) which are downstream signaling components of ErbB receptors [39]. Specific anti phospho-protein antibodies to Erk1/2 and JNK were used. As shown in Figure 4, NRG induced an increase in the phosphorylation of Erk1/2 and JNK. NAC, which inhibits NRG-induced autophagy, had no effect on Erk1/2 phosphorylation, indicating that Erk activation is not involved in NRG-induced autophagy or that NAC acts downstream to Erk activation. On the other hand, JNK phosphorylation was strongly reduced in the presence of NAC, indicating that JNK may be a potential mediator of NRG-induced autophagy.

**Figure 5:**
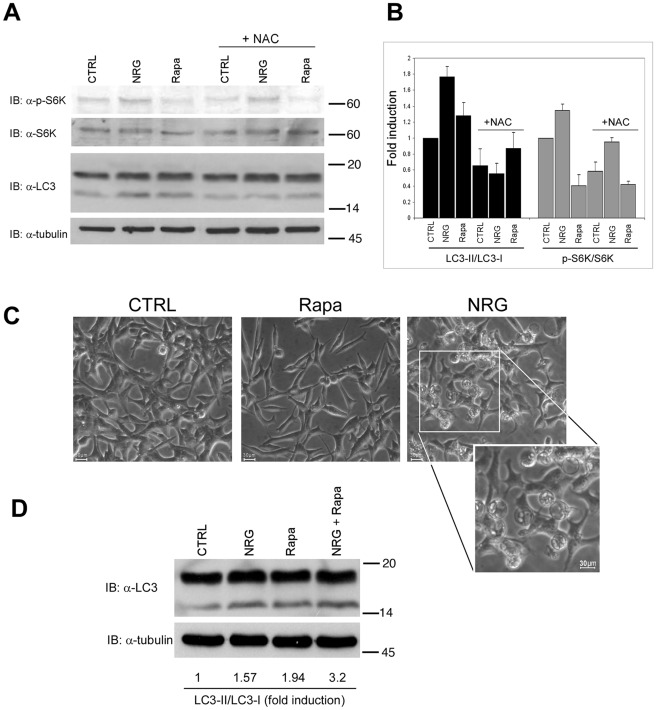
Analysis of NRG-induced autophagy compared to Rapamycin-mediated autophagy. (A) LNCaP cells were treated with either 100 ng/ml NRG or 50 nM rapamycin (Rapa) for 24 h, in the presence or in the absence of 10 mM NAC. Whole cell lysates were prepared and subjected to an immunoblot analysis with anti-LC3, anti-phospho-S6K and anti-S6K antibodies. (B) Densitometric analysis of the results described in A is represented as fold induction of the control untreated cells (n = 3, means ± S.D). (C) Representative images of cell morphology following treatments with NRG and rapamycin are shown (Olympus, 20×magnification). (D) LNCaP cells were treated with either 100 ng/ml NRG, 50 nM rapamycin or both for 24 h. Whole cell lysates were prepared and subjected to an immunoblot analysis with anti-LC3 antibodies. This experiment was repeated three times with similar results.

Since NRG induced S6K phosphorylation but also induced autophagy, we next compared autophagy induced by mTOR inhibition to the autophagy induced by NRG. The mTOR inhibitor, rapamycin, was previously shown to induce autophagy by downregulating mTOR activity [37], [40]. We found that rapamycin treatment as well as NRG treatment induced autophagy, as judged by the increased LC3-II/LC3-I ratio (Figure 5A and B) and by enhanced LC3 puncta formation (Figure 1B). However, as expected, S6K phosphorylation was increased following NRG treatment but reduced following rapamycin treatment. In addition, NAC treatment inhibited autophagy induced by rapamycin and NRG, however it had no effect on NRG-induced S6K phosphorylation. Interestingly, rapamycin treatment although inhibited cell growth [29], had no effect on cells morphology, while NRG caused a dramatic morphological change as previously described [28], [29] and as demonstrated in Figure 5C and Figure S1 (round and detached cells). These results demonstrate that NRG-induced autophagy differs from autophagy triggered by rapamycin. Next, we examined the effect of NRG and rapamycin co-treatment on autophagy-inducion in LNCaP cells (Figure 5D). As shown, combined treatment induced higher levels of LC3II compared to each treatment alone, suggesting that rapamycin and NRG might act through different signaling pathways to induce autophagy.

### NRG-induced Autophagic Cell Death in LNCaP Cells Depends on JNK Activation

**Figure 6:**
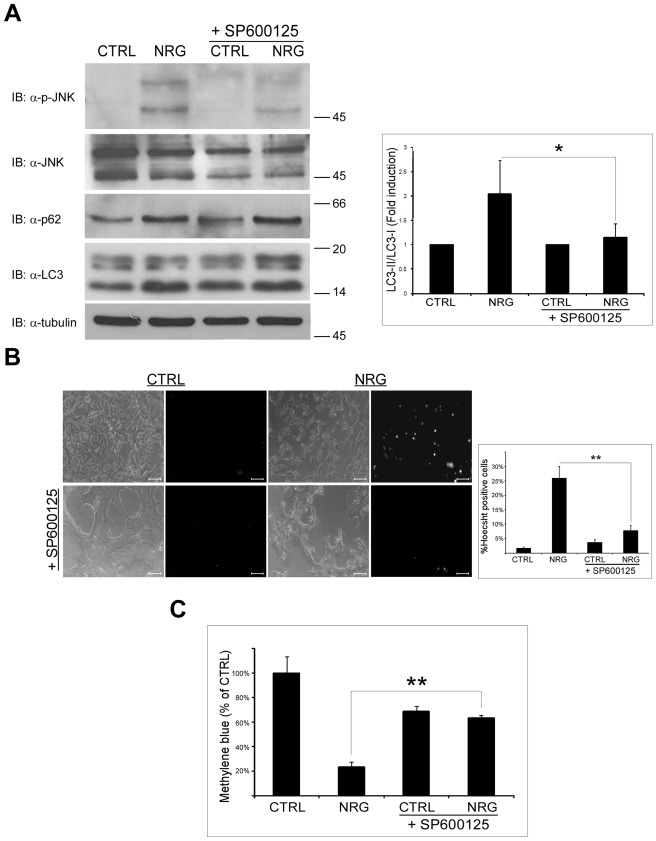
SP600125 inhibits NRG-induced autophagy and cell death. (A) LNCaP cells were treated with 100 ng/ml NRG for 24 h with or without 20 µM SP600125. Whole cell lysates were prepared and subjected to an immunoblot analysis with anti-LC3, anti-p62, anti-p-JNK and anti-JNK antibodies. *Left panel,* representative results. *Right panel,* densitometric analysis is presented as fold induction over the control untreated cells (n = 5; means ± S.D; *p<0.05). (B) LNCaP cells were treated with 100 ng/ml NRG in the presence or in the absence of 20 µM SP600125 for 60 h. The cells were stained with the fluorescent DNA dye bisbenzimide (Hoecsht 33258, 1 µg/ml) to assess the number of dying cells. Following staining, the cells were photographed using Olympus optical inverted phase-contrast microscope Model IX70 (20×magnification; scale bar, 50 micrometer). *Left panel*, representative images are shown. *Right panel*, percentage of dying cells was estimated by counting the number of Hoecsht-positive cells compared to the number total cells in each field (10–15 fields for each treatment, 100–200 cells per field). Results are presented as mean ± S.D (**p<0.0001). (C) LNCaP cells were tested for cell viability using the methylene blue staining assay. Cells were treated with 100 ng/ml NRG in the presence or in the absence of 20 µM SP600125. Methylene blue assay was performed 60 h later. Results are presented as % of control, and are the mean ± S.D of 4–6 determinations (**p<0.0001). This experiment was repeated three times with similar results.

Because mTOR pathway is not involved in NRG-induced autophagy and cell death, we searched for other possible mediators. We chose to examine the involvement of JNK, since NRG induced JNK phosphorylation, which was inhibited by NAC. Therefore, we first examined the effect of JNK inhibitor SP600125 on NRG-induced autophagy (Figure 6A). As shown, in the presence of SP600125, NRG-induced autophagy was inhibited, as judged by the decreased LC3-II/LC3-I ratio. Yet, SP600125 treatment had no effect on p62 levels. Next, we examined the effect of JNK inhibitor on cell viability using Hoecsht dye exclusion and methylene blue staining assays (Fig. 6B and C, respectively). As shown, in the presence of JNK inhibitor, NRG-induced cell death was inhibited; indicating that JNK activation by NRG may be important for the induction of autophagy and cell death of LNCaP cells.

### Beclin 1 is Necessary for and Bcl-2 Confers Resistance to NRG-induced Autophagy and Cell Death

**Figure 7:**
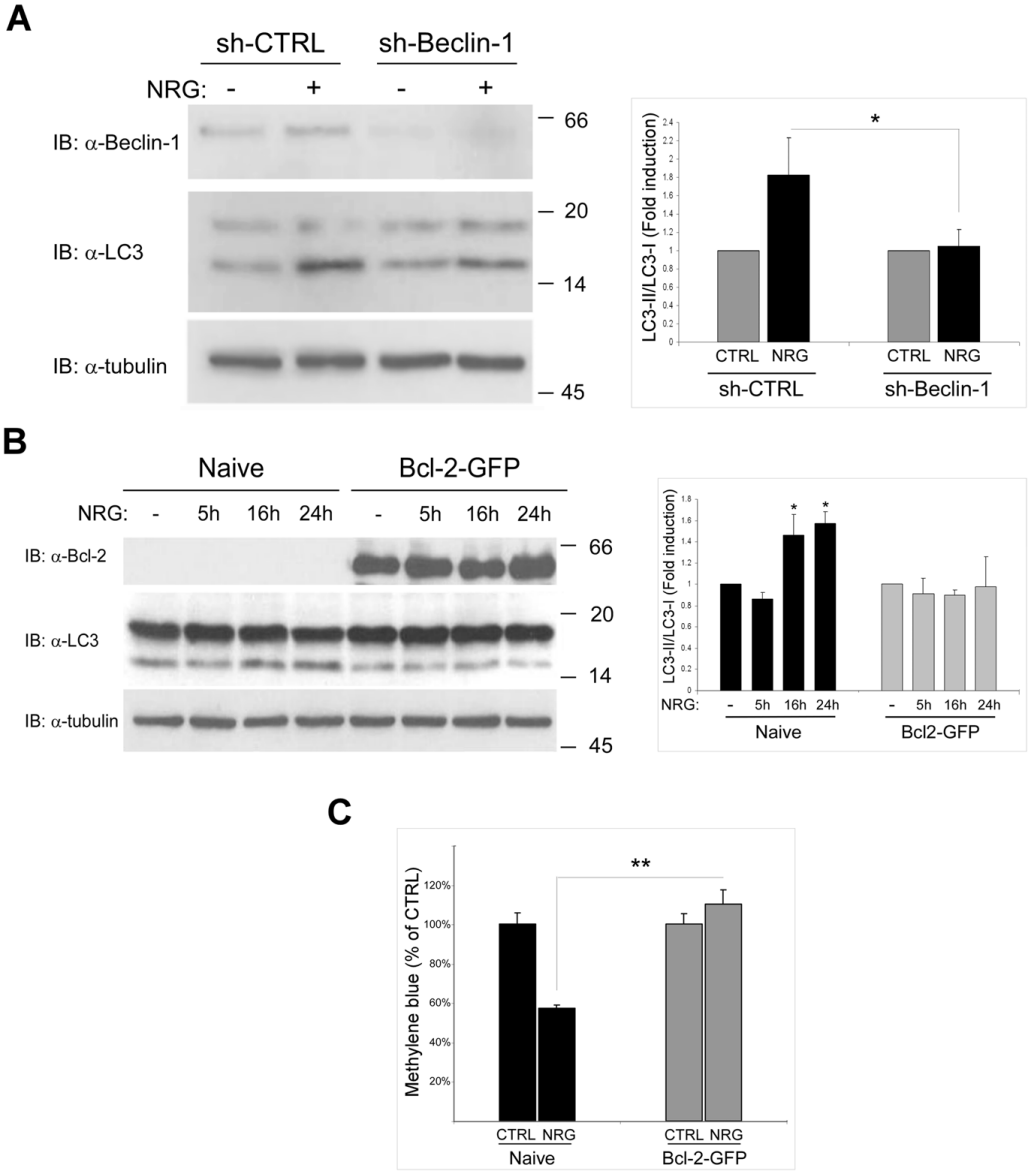
NRG-induced autophagy is Beclin-1 dependent. (A) LNCaP cells were transfected with Beclin 1 sh-RNA (3 µg) or control scrambled sh-RNA (3 µg) and incubated in normal medium for 72 h. The cells were then treated with 100 ng/ml NRG for additional 24 h. Whole cell lysates were prepared and subjected to an immunoblot analysis with anti-LC3 and anti-Beclin 1 antibodies. *Left panel*, representative experiment is shown. *Right panel*, quantification of the results is shown. The results are presented as fold induction compared to the control untreated cells (n = 3; means ± S.D; *p<0.05). (B) Naïve or Bcl-2-GFP stably expressing LNCaP cells were treated with 100 ng/ml NRG for the indicated time periods. Whole cell lysates were prepared and subjected to an immunoblot analysis with anti-LC3 and anti-Bcl-2 antibodies. *Left panel*, representative blot is shown. *Right panel*, quantification of the results is presented as fold induction compared to the control untreated cells (n = 3; means ± S.D; *p<0.05). (C) Naïve and Bcl-2-GFP stably expressing LNCaP cells were tested for cell viability using the methylene blue staining assay. Cells were treated with 100 ng/ml NRG and the methylene blue assay was performed 60 h later. Results are presented as % of control, and are the mean ± S.D of 4–6 determinations (**p<0.0001). This experiment was repeated three times with similar results.

Beclin 1, a component of the class-III PI3K complex, is a major known regulator of autophagy [41], [42]. To determine the involvement of this pathway in NRG-induced autophagy, LNCaP cells were transfected with sh-Beclin 1 or control scrambled sh-RNA expression vectors. As shown in Figure 7A, NRG treatment enhanced autophagy in the control cells. However in sh-Beclin 1 transfected cells, NRG-mediated autophagy was reduced compared to the control cells. These results indicate that Beclin 1 protein is involved in NRG mediated autophagy in LNCaP cells.

Members of the Bcl-2 anti-apoptotic family were previously shown to inhibit the autophagy-promoting activity of Beclin 1 [23]. It was also demonstrated that during autophagy the interaction between Bcl-2 anti-apoptotic proteins and Beclin 1 is inhibited. Assuming that Beclin 1 is involved in NRG-induced autophagy and cell death, we hypothesized that overexpression of Bcl-2 would protect LNCaP cells from NRG-induced autophagy and cell death. Thus, we next examined the effect of Bcl-2-GFP overexpression on NRG-mediated autophagy of LNCaP cells (Figure 7B). As shown, autophagy in naive LNCaP cells was increased after 16 h and 24h of NRG treatment, while in LNCaP cells stably overexpressing Bcl-2-GFP, NRG treatment had no effect on the levels of autophagy induction. Furthermore, Bcl-2-GFP overexpressing LNCaP cells were resistant to NRG-induced cell death compared to naive LNCaP cells (Fig. 7C). Taken together, our results strongly suggest that in addition to JNK activation, Beclin 1 is also essential for the autophagic process promoted by NRG.

## Discussion

Prostate cancers typically start as androgen-sensitive lesions but frequently develop into androgen-insensitive lesions with the progression to advanced stages. The LNCaP androgen-dependent cell line expresses high levels of ErbB-2 and ErbB-3 compared to other human prostate cancer cells [28]. In addition, these cells do not express NRG but they do express TGF-α and EGF, which can function as autocrine activators of the EGFR [28]. Previously, it was demonstrated that in LNCaP cells grown without androgen mimetic, NRG but not EGF induces cell death. This effect of NRG on cell death is mediated by ErbB-2/ErbB-3 heterodimers [29]. It was also demonstrated that the cell death induced by NRG is inhibited by PI3K inhibitors, indicating that NRG may induce autophagic cell death [29]. In the present study, we demonstrate for the first time that NRG indeed induces autophagy in LNCaP cells, using two assays: immunoblot analysis with anti-LC3 antibodies and microscopic analysis of LC3-GFP staining in LNCaP cells. This effect of NRG was also inhibited by 3-MA. However, the autophagy induced by NRG is incomplete since no degradation of p62 protein following NRG treatment was detected. Thus, NRG activating ErbB2/ErbB3 heterodimers induces incomplete autophagy and cell death in LNCaP cells.

Reactive oxygen species (ROS) were implicated in the signaling pathways initiated by receptor tyrosine kinases, including ErbB receptors [43], [44]. Several line of evidence demonstrates that reactive oxygen species (ROS) play a role in autophagy. First, ROS are required for starvation-induced autophagy, apparently due to regulation of Atg4 activity [32]. Second, ROS themselves can induce autophagy in certain cell lines [31], [45]. Our results indicate that NRG-induced autophagy is sensitive to ROS levels, since in the presence of the general antioxidant NAC, NRG-induced autophagy and cell death were inhibited.

It was previously shown that mTOR serves as a negative regulator of autophagy by suppressing the activity of Atg13/Atg1 complex [46]. Thus, growth factors and certain hormones exert their anti-autophagic effect by activating the class-I PI3K/Akt/mTOR pathway [47]. On the other hand, pro-autophagic stimuli, such as nutrients starvation and rapamycin treatment, lead to mTOR inactivation followed by autophagy induction. Indeed, inhibition of mTOR by rapamycin induced autophagy in LNCaP cells. On the same cells, our results demonstrate that NRG induces mTOR activation as evident by the phosphorylation of S6K, and its upstream regulator Akt. In addition, it was previously demonstrated that NRG induces ErbB2/ErbB3 heterodimer formation and class-I PI3K activation in LNCaP cells [28]. Taken together, it appears that NRG activates an anti-autophagic signaling pathway, namely the ErbB/class-I PI3K/Akt/mTOR pathway and yet promotes autophagy induction in LNCaP cells. NAC, which completely blocks the NRG-induced autophagy, did not affect the phosphorylation of neither Akt nor S6K. Hence, we suggest that NRG-induced autophagy is independent of mTOR inhibition.

NRG induces activation of various signaling pathways. It was previously shown that in LNCaP cells, NRG activates among other signaling pathways also the MAP kinases: Erk, p38, JNK and the PI3K signaling pathways [28]. We attempted to follow the signaling pathway leading to NRG induced autophagy and cell death. Our results indicate that JNK is involved in NRG-induced autophagy and cell death. Indeed, increasing body of evidence exists regarding the role of JNK as a mediator of autophagy induction following various stimuli [48], [49], [50]. In agreement with these findings, we showed that JNK phosphorylation increases following NRG treatment in LNCaP cells. We also found that NAC, an inhibitor of NRG-induced autophagy and cell death, blocks JNK phosphorylation. Taken together, we suggest that JNK mediates the pro-autophagic effect of NRG. Indeed, in the presence of JNK inhibitor SP600125, NRG-induced cell death and autophagy were inhibited.

The nucleation and assembly of the autophagosome requires activation of class-III PI3K complex, which is composed of the PI3K (vps34), vps15, atg14 and atg6 (Beclin 1 in mammalian cells) [19], [20]. Beclin 1-mediated autophagy is negatively regulated by its interaction with Bcl-2 anti apoptotic proteins. Upon autophagy initiation, the interaction between Bcl-2 and Beclin 1 is inhibited, the class-III PI3K complex turns active and autophagy is promoted [23]. Our results support the involvement of this pathway in NRG-mediated autophagy induction. First, we demonstrated that NRG-induced autophagy is blocked by 3-MA, an inhibitor of class-III PI3K complex. Second, upon silencing of Beclin 1, the autophagy induced by NRG treatment decreases. Finally, we found that Bcl-2 overexpression inhibits NRG-dependent autophagy and cell death. Several studies proposed that JNK-mediated Bcl-2 phosphorylation induces the dissociation of Bcl-2 from Beclin 1, thus promoting autophagy [49], [50]. Therefore, it might be that NRG exerts its pro-autophagic effect through inhibition of the interaction between Beclin 1 and Bcl-2 anti apoptotic proteins.

In summary, we demonstrated that NRG induces incomplete autophagy and cell death of LNCaP cells. The induction of autophagy by NRG is mediated via JNK and Beclin 1 signaling pathways. The autophagy induced by NRG is mTOR-independent. The autophagy and cell death induced by NRG can be blocked by JNK inhibition, Bcl-2 overexpression, Beclin 1 downregulation and NAC, indicating the requirement of JNK, Beclin 1 and ROS for the process. Since inhibition of NRG induced autophagy rescue from cell death, we suggest that the incomplete autophagy may be linked to the observed cell death.

## Materials and Methods

### Materials and Buffers

Antibodies were obtained from the following sources: monoclonal mouse anti Bcl-2 (Santa Cruz Biotechnology, sc-7382), polyclonal rabbit anti Beclin 1 (Santa-Cruz Biotechnology, sc-11427), polyclonal rabbit anti Erk2 (Santa-Cruz Biotechnology, sc-154), polyclonal rabbit anti Akt (Santa-Cruz Biotechnology, sc-8312), monoclonal mouse anti p62/SQSTM1 (Santa-Cruz Biotechnology, sc-28359), monoclonal mouse anti β-tubulin1 (Sigma, T7816), polyclonal rabbit anti LC3B (Sigma, L7543), polyclonal rabbit anti phospho-Thr389-S6 kinase (Sigma, S6311), polyclonal rabbit anti S6 kinase (Sigma, S4047), monoclonal mouse anti phospho-Erk1/2 (Sigma, 8159), polyclonal rabbit anti JNK (Cell Signaling, 9252), polyclonal rabbit anti phospho-Ser473-Akt (Cell Signaling, 9271), polyclonal rabbit anti phospho-Thr183/Tyr185-JNK (Cell Signaling, 9251), monoclonal mouse anti caspase 9 (Cell Signaling, 9508). Proteins and reagents are as follows: human recombinant NRG (NRG, R&D System Inc. 396-HB/CF), Bafilomycin A_1_ (Sigma, B1793), Rapamycin (Calbiochem, 553210), 3-methyladenine (3-MA, Sigma, M9281), *N*-acetylcycteine (NAC, Sigma, A8199), SP600125 (Sigma, S5567), EBSS (Sigma, E3024).

### Cell Line

The human LNCaP prostate cell line was obtained from American Type Culture Collection, MA, USA. Cells were grown in RPMI-1640 (Biological Industries, 01-100-1) supplemented with antibiotics and 10% heat-inactivated fetal bovine serum (FBS, Hyclone, CH30160.03). Cells were incubated at 37^o^C in 5% CO_2_ in air, and the medium was changed every 3–4 days. Cells were passaged when 70% confluent using trypsin/Di-sodium ethylenediaminetetra-acetic acid (Biological Industries, 03-045-1). Two days before each experiment, cells were cultured at 30% confluence in phenol red-free RPMI-1640 (Biological Industries, 01-104-5) supplemented with 5% dextran-coated charcoal-stripped FBS (Biological Industries, 04-201-1).

### Stable and Transient Transfections

For stable transfections, the jet-PEI reagent was used according to the manufacturer's instructions (Polyplus transfection, 101–10). In brief, LNCaP cells were incubated in phenol red-free RPMI-1640 supplemented with 5% dextran-coated charcoal-stripped FBS and containing 3 µg plasmid DNA and 6 µl jet-PEI reagent for overnight. Then, the medium was replaced with fresh RPMI-1640 supplemented with 10% FBS. Stable clones expressing LC3-GFP or Bcl-2-GFP were selected and cultured in 400 µg/ml geneticin (G-418, Calbiochem, 345810). sh-RNA for human Beclin 1 was gifted from Prof. Adi Kimchi (Weizmann Institute, Israel). For transient transfections with sh-RNA against human Beclin 1, the Lipofectamine 2000 reagent was used (Invitrogen, 11668-019). The cells were incubated with 4 µg plasmid DNA and 8 µl transfection reagent for 8 h, and then the medium was replaced.

### Cell Survival Assays

Cells were plated at 10^5^ cells/ml, in phenol red-free RPMI-1640 supplemented with 5% dextran-coated charcoal-stripped FBS and treated without or with 100 ng/ml NRG in the presence or in the absence of the indicated drugs for 60 h. Cell viability was determined by methylene blue assay. The cells were fixed with 4% formaldehyde in phosphate buffered saline (PBS) for 2 h, washed once with 0.1 M boric acid (pH = 8.5) and incubated with the DNA-binding dye methylene blue (1% in boric acid; Sigma, M9140) for 20 min at room temperature. Cells were washed three times and then lysed using 0.1 M HCl. Absorbance was measured at 595 nm using the Tecan Spectrafluor Plus spectrophotometer. Cell viability was calculated as the ratio of absorbance in the treated cultures compared to the control untreated cultures. Staining of nuclei with the fluorescent DNA dye bisbenzimide, (Hoechst 33258) was used to estimate the number of dying cells. Hoechst staining was performed on live cells by incubation with Hoechst solution (1 µg/ml; Sigma, B2883) for 10 min. Following staining, cells were photographed using the Olympus optical inverted phase-contrast microscope Model IX70 (×20 magnification). Nuclear staining and nuclear morphology scored dead cells. Percentage of dead cells was estimated by calculating the number of nuclei stained with Hoecsht 33258 compared to the total cells number in each field.

### Cell Cycle Analysis

For cell cycle analysis, cells were seeded at 10^5^ cells/ml in phenol red-free RPMI-1640 supplemented with 5% dextran-coated charcoal-stripped FBS. The cells were treated as indicated for 60 h. After which, 10^6^ cells were washed once with PBS, permeabilized using 0.1% triton X and stained with 50 µg/ml propidium iodide (Sigma, P4170). The stained cells were analyzed in a fluorescence-activated cell sorter (FACScan; Becton and Dickinson) within 30 min. the percentage of cells at different stages of the cell cycle was determined using the WinMDI 2.9 program.

### Lysate Preparation and Immunoblotting

Cells were grown for 48 h in serum free phenol red-free RPMI-1640 and then were exposed to the indicated stimuli. After treatment, cells were solubilized in lysis buffer (50 mM HEPES pH = 7.5, 150 mM NaCl, 10% glycerol, 1% triton X, 1 mM EDTA pH = 8, 1 mM EGTA pH = 8, 1.5 mM MgCl_2_, 200 µM Na_3_VO_4_, 150 nM aprotinin, 1 µM leupeptin, 500 µM AEBSF). Lysates were cleared by centrifugation and a boiling gel sample buffer was added to cell lysates. Lysates were resolved by SDS-polyacrylamide gel electrophoresis through 7.5–12.5% gels and electrophoretically transferred to nitrocellulose membrane. Membranes were blocked for 1 h in TBST buffer (0.05 M Tris HCl pH 7.5, 0.15 M NaCl and 0.1% Tween 20) containing 6% milk, blotted with primary antibodies for 2 hours, followed by secondary antibody linked to horseradish peroxidase for 1 h. Immunoreactive bands were detected with the enhanced chemiluminescence reagent.

### Statistical Analysis

All experiments were performed at least three times. Results are presented as mean ± SD. One-tailed Student's t-test was used to assess the differences between means. Results were considered statistically significant when P-value was <0.05.

## Supporting Information

Video S1 **Morphological changes induced by NRG over 20 h treatment.** LNCaP cells were treated with 100 ng/ml neuregulin. After 2 h of treatment, 25 mM HEPES was added to the growth medium and the cells were observed for additional 20 h in 37°C using time-lapse phase-contrast microscopy (the method is described in Materials and Methods S1).
(WMV)

Figure S1 **LC3 and p62 levels after starvation of LNCaP cells.** LNCaP cells were incubated with RPMI-1640 10% FCS for 24 h (CTRL) or with EBSS medium for the indicated time period. Whole cell lysates were prepared and subjected to an immunoblot analysis with anti-LC3 and anti-p62 antibodies.
(TIF)

Materials and Methods S1 (DOC)
