# Supplementary figures and images for: Neuregulin Promotes Incomplete Autophagy of Prostate Cancer Cells That Is Independent of mTOR Pathway Inhibition
Source: PLoS One. 2012 May 14;7(5):e36828. doi: 10.1371/journal.pone.0036828 (PMC3351469; doi:10.1371/journal.pone.0036828)

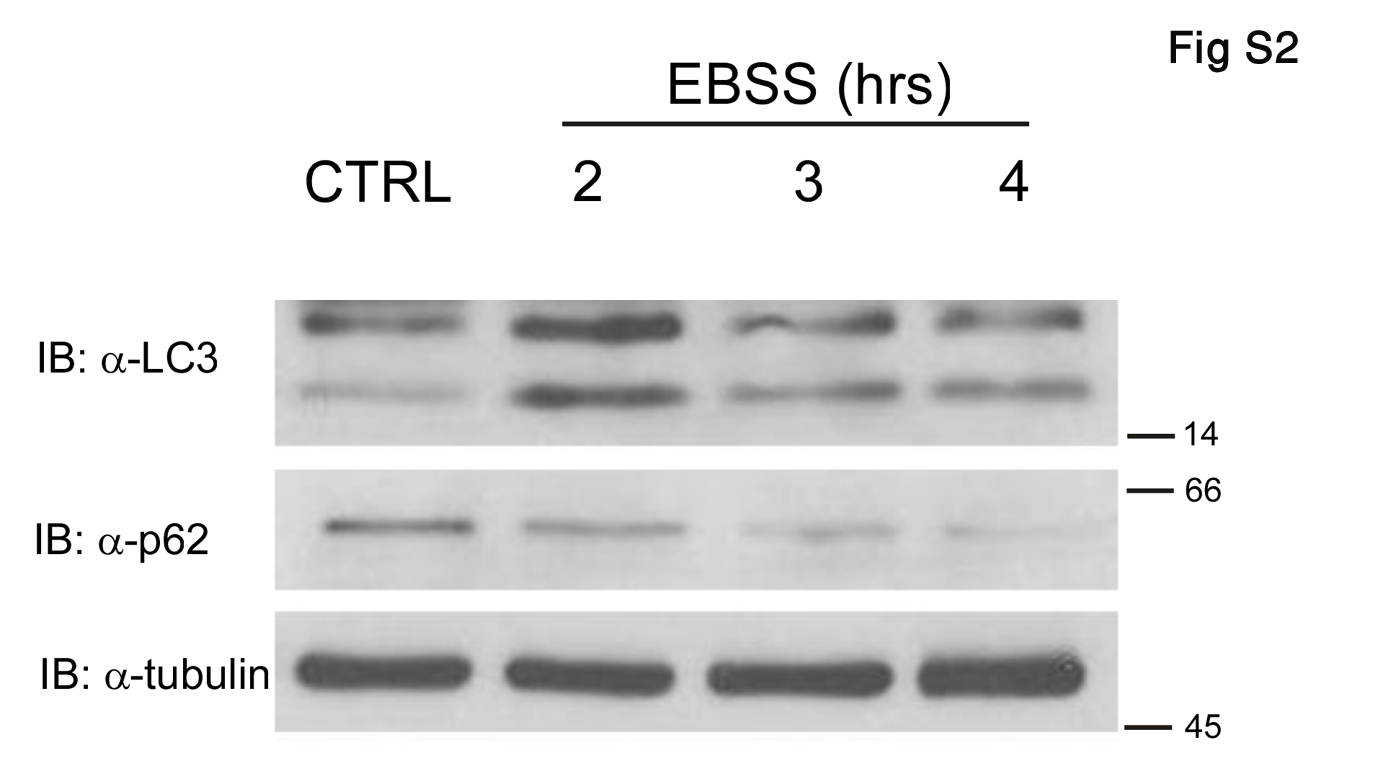

Supplement: Figure S1 — LC3 and p62 levels after starvation of LNCaP cells. LNCaP cells were incubated with RPMI-1640 10% FCS for 24 h (CTRL) or with EBSS medium for the indicated time period. Whole cell lysates were prepared and subjected to an immunoblot analysis with anti-LC3 and anti-p62 antibodies. (TIF) [file pone.0036828.s002.tif]
