# Supplementary material for: Neuregulin Promotes Incomplete Autophagy of Prostate Cancer Cells That Is Independent of mTOR Pathway Inhibition
Source: PLoS One. 2012 May 14;7(5):e36828. doi: 10.1371/journal.pone.0036828 (PMC3351469; doi:10.1371/journal.pone.0036828)
Supplement: Materials and Methods S1 — (DOC) [file pone.0036828.s003.doc]

**Materials and methods S1**

*Live cell imaging* - For in vivo time-lapse studies, LNCaP cells were cultured with RPMI-1640 medium without phenol red, supplemented with 5% charcoal stripped FBS for 48 h. At this point, the cells were treated with 100 ng/ml NRG. Morphological changes were followed 2 h after treatment has begun for another 20 h. Growth medium was supplemented with 25 mM HEPES and the cells were observed in 37°C using time-lapse phase-contrast microscopy (Axiovert 200M, Carl Zeiss MicroImaging under control of SlideBook™ software, Intelligent Imaging Innovations; 10x Zeiss Plan Neofluar; CoolSnap EZ, Photometrics). Images were acquired at 4 min intervals. Time lapse sequences are presented as movies, at 40 frames per second.
